# The acute effects of lower limb intermittent negative pressure on foot macro- and microcirculation in patients with peripheral arterial disease

**DOI:** 10.1371/journal.pone.0179001

**Published:** 2017-06-07

**Authors:** Øyvind Heiberg Sundby, Lars Øivind Høiseth, Iacob Mathiesen, Harald Weedon-Fekjær, Jon O. Sundhagen, Jonny Hisdal

**Affiliations:** 1 Section of Vascular Investigations, Department of Vascular Surgery, Oslo University Hospital, Oslo, Norway; 2 Faculty of Medicine, Institute of Clinical Medicine, University of Oslo, Oslo, Norway; 3 Otivio AS, Oslo, Norway; 4 Department of Anesthesiology, Oslo University Hospital, Oslo, Norway; 5 Oslo Center for Biostatistics and Epidemiology, Research Support Services, Oslo University Hospital, Oslo, Norway; 6 Department of Vascular Surgery, Oslo University Hospital, Oslo, Norway; Medical University Innsbruck, AUSTRIA

## Abstract

**Background:**

Intermittent negative pressure (INP) applied to the lower leg and foot increases foot perfusion in healthy volunteers. The aim of the present study was to describe the effects of INP to the lower leg and foot on foot macro- and microcirculation in patients with lower extremity peripheral arterial disease (PAD).

**Methods:**

In this experimental study, we analyzed foot circulation during INP in 20 patients [median (range): 75 (63-84yrs)] with PAD. One leg was placed inside an air-tight vacuum chamber connected to an INP-generator. During application of INP (alternating 10s of -40mmHg/7s of atmospheric pressure), we continuously recorded blood flow velocity in a distal foot artery (ultrasound Doppler), skin blood flow on the pulp of the first toes (laser Doppler), heart rate (ECG), and systemic blood pressure (Finometer). After a 5-min baseline sequence (no pressure), a 10-min INP sequence was applied, followed by 5-min post-INP (no pressure). To compare and quantify blood flow fluctuations between sequences, we calculated cumulative up-and-down fluctuations in arterial blood flow velocity per minute.

**Results:**

Onset of INP induced an increase in arterial flow velocity and skin blood flow. Peak blood flow velocity was reached 3s after the onset of negative pressure, and increased 46% [(95% CI 36–57), *P*<0.001] above baseline. Peak skin blood flow was reached 2s after the onset of negative pressure, and increased 89% (95% CI 48–130), *P*<0.001) above baseline. Cumulative fluctuations per minute were significantly higher during INP-sequences compared to baseline [21 (95% CI 12–30)cm/s/min to 41 (95% CI 32–51)cm/s/min, *P*<0.001]. Mean INP blood flow velocity increased significantly ~12% above mean baseline blood flow velocity [(6.7 (95% CI 5.2–8.3)cm/s to 7.5 (95% CI 5.9–9.1)cm/s, *P* = 0.03)].

**Conclusion:**

INP increases foot macro- and microcirculatory flow pulsatility in patients with PAD. Additionally, application of INP resulted in increased mean arterial blood flow velocity.

## Introduction

Lower extremity atherosclerotic peripheral arterial disease (PAD) results in reduced tissue perfusion and inadequate oxygen delivery due to narrowing of the arterial tree [1]. Insufficient blood flow may result in inadequate nutritive supply with tissue breakdown and ulceration, a process that if untreated may lead to frank gangrene and loss of the extremity [1]. The majority of treatment strategies for PAD are geared towards impeding progression of the disease and increasing blood flow [1]. The first-line treatment for PAD focuses on behavioral changes, such as reducing risk factors and improving exercise performance through ambulatory exercise [1–3]. If behavioral changes fail, the next line of therapy is revascularization through endovascular or open surgery [4]. Unfortunately, the current treatment strategies are insufficient for many PAD patients [5–7].

We have recently demonstrated that intermittent negative pressure (INP) applied to the lower leg and foot increased arterial and skin blood flow in the foot in healthy volunteers, with only minor changes in central hemodynamics [8]. Using INP with short negative pressure (-40mmHg) pulses lasting 10 to 30s, the increase in flow was due to INP-induced increases in flow pulsatility [8]. Furthermore, an eight-week pilot study on four PAD patients with hard-to-heal lower limb ulcers indicated that repetitive use of INP may improve long-term clinical outcomes, such as wound healing and foot perfusion [9]. Despite promising results for the use of INP to increase peripheral circulation in healthy volunteers, the acute effects of INP on PAD patients’ peripheral circulation are unknown.

The aim of the present study was therefore to describe the effects of applying INP to the lower leg and foot on foot macro- and microcirculation and central hemodynamics in patients with PAD. Based on our previous findings in healthy volunteers [8], we hypothesized that INP would increase flow pulsatility in the foot and increase arterial and skin blood flow.

## Materials and methods

Eligible PAD patients were recruited from the outpatients’ clinic at Section of Vascular Investigations, Oslo University Hospital, Aker between October 2015 and April 2016. The experimental protocol was approved by the *Regional Committees for Medical and Health Research Ethics* in Norway (protocol number: 2014/1967) and performed in accordance with the Declaration of Helsinki. Written and oral informed consent was obtained from all patients before the experiment began.

### Participants

The twenty-one patients recruited into the study had symptomatic lower extremity PAD (Fontaine stage grade II [1], with obstructions of the aortoiliac (n = 2) and femoropopliteal (n = 19) arteries, and an ankle-brachial pressure index (ABPI) at rest of ≤0.9. Exclusion criteria were: i) chronic respiratory insufficiency or ii) eczema and psoriasis or iii) uncontrolled hypertension or iv) recent (12 months) vascular, abdominal, cardiothoracic, or lower limb orthopedic surgery.

All patients refrained from eating two hours before the experiment and from using alcohol, tobacco or caffeine on the experimental day.

### Anthropometric and diagnostic measurements

We measured the patients’ height and weight before a 5-min rest. Thereafter, we obtained their supine resting ABPI [10]. The limb with the lowest systolic ankle pressure was chosen as the test leg during the experiment (limb exposed to INP). The pulse volume recording curve was measured in both lower limbs with an air-plethysmography cuff placed on each subject’s distal ankle (Stranden MacroLab, STR Teknikk, Aalesund, Norway). The amplitude correlates with arterial inflow to the extremity, and a sequential reduction in pulse wave amplitude from the thigh to the calf signifies the presence of a flow-limiting lesion in the more proximal arterial segment [11].

The patients’ exercise tolerance was tested through an exercise treadmill test using a Skinner-Gardner protocol [12] on a treadmill (Lexco, LGT 7716S, Taeyoung Co, Korea). Briefly, the protocol uses a progressive graded workload with a constant speed of 3.2 km/h, and an increased grade of 2% every 2-min [12]. The maximal walking distance was recorded.

### Experimental design

**Fig 1:**
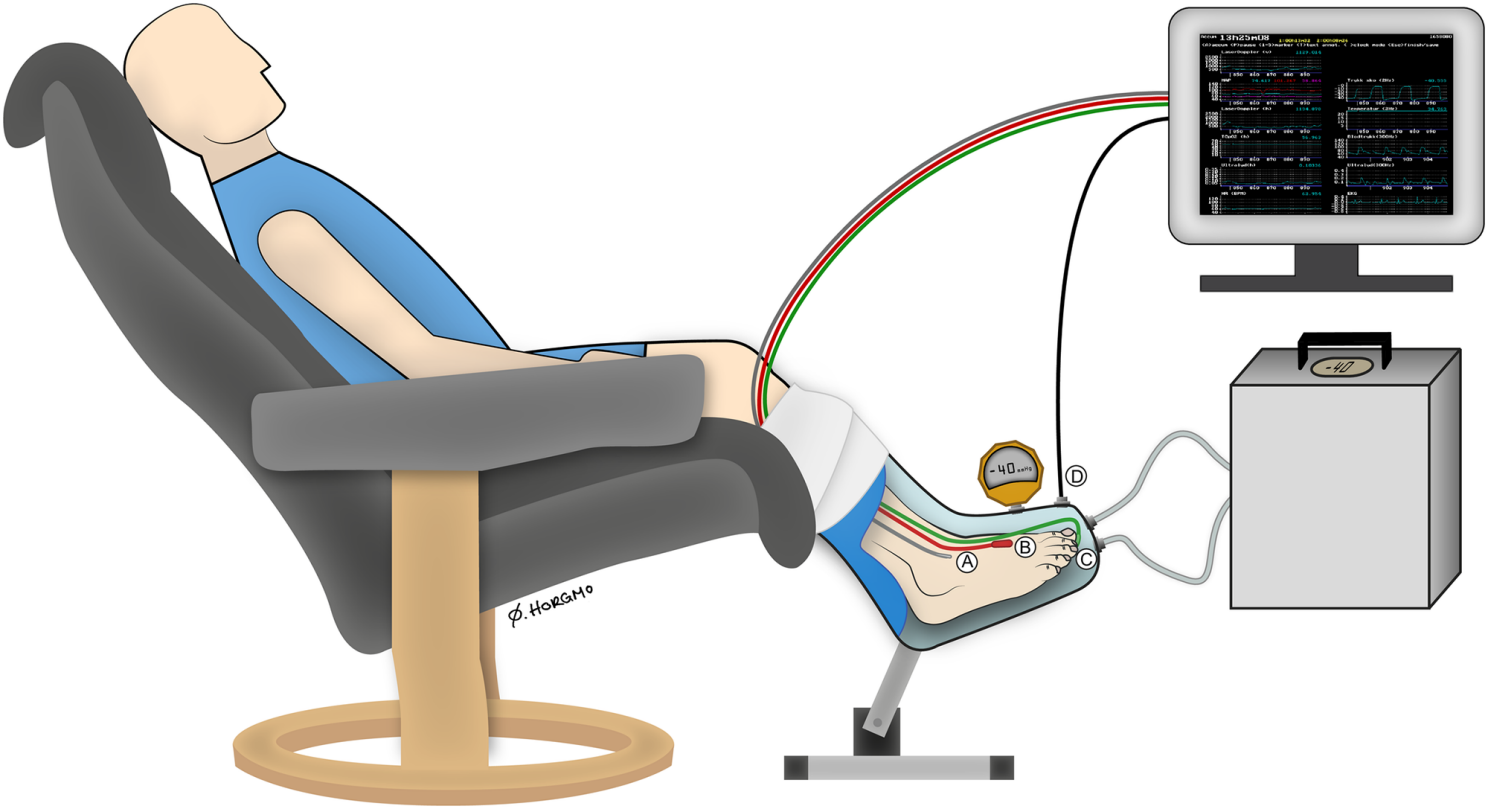
Illustration of the experimental setup with probes attached to the foot. The patient’s test leg was placed in the custom-made vacuum chamber interfaced with the pressure control system. The vacuum chamber was sealed around the patient’s leg below the knee. The contralateral leg was placed outside the vacuum chamber, acting as a control in atmospheric pressure. A: Skin temperature probe; B: Ultrasound Doppler probe; C: Laser Doppler flux probe; D: Pressure transducer from vacuum chamber interfaced with the computer. An additional external calibrated pressure gauge (Fluke, 700G Series, Everett, WA, USA) was used for calibration (REGIST 3). Illustration: Øystein H. Horgmo, University of Oslo.

The patients were comfortably clothed and seated in an armchair for 20-min before the experiment started. After attaching the probes to the foot (Fig 1), the patients’ feet were covered with loose, non-elastic wool socks to keep them under thermoneutral ambient conditions. A light blanket covered the patient’s body to avoid cooling.

**Fig 2:**
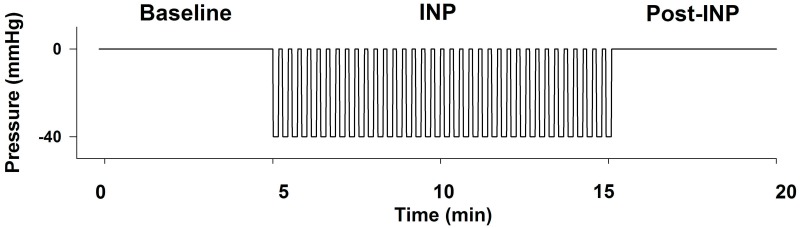
Description of the study sequences and the intermittent negative pressure (INP) applied. The three sequences were as follows: (i) 5-min baseline (no pressure), (ii) 10-min INP, followed by (iii) 5-min post-INP (no pressure). One pressure cycle equals 10s -40mmHg negative pressure and 7s atmospheric pressure. INP = intermittent negative pressure.

During the experiment, the patients were asked to sit with an approximate angle of 130° in their knee and hip joints (Fig 1). The test leg was placed in a rigid molded polyethylene custom-made vacuum chamber coupled to a pressure control system (FlowOx^™^; Otivio AS, Oslo, Norway). The vacuum chamber had internal padding to allow insertion of a leg with probes and prevent pressure points on the leg and skin. Only the posterior part of the lower leg touched the padding (Fig 1). The vacuum chamber was sealed just below the knee with a thermoplastic elastomer (TPS-SEBS) to allow for application of negative pressure. The contralateral leg was placed outside the vacuum chamber in atmospheric pressure. Pressures were continuously monitored throughout the study using a calibrated pressure probe attached to the vacuum chamber. The experimental sequences are shown in Fig 2. Briefly, 5-min baseline registrations were performed with no pressure manipulation (atmospheric pressure). Thereafter, INP was applied for 10-min using an oscillation protocol with cycles of 10s -40mmHg negative pressure and 7s atmospheric pressure [8]. Finally, a 5-min post-INP sequence at atmospheric pressure was recorded. All experiments were conducted in a quiet and temperature-controlled environment (25.1±1.5°C) to allow patients to obtain their thermoneutral zone and to reduce sympathetic stress that could create artifacts [13]. Data were analyzed in custom-made software (REGIST 3, Morten Eriksen, University of Oslo, Oslo, Norway).

### Signal acquisition and analysis

Arterial blood flow velocity was measured continuously in the dorsalis pedis/posterior tibial artery with a 10MHz pulsed Doppler probe (SD-50; GE Vingmed Ultrasound, Horten, Norway). The ultrasound beam was positioned centrally at either the dorsum pedis or posterior to the medial malleolus of the ankle, and attached with surgical adhesive tape (Micropore Surgical Tape, 3M, MN, US). If it was not possible to acquire a flow signal in a patient’s dorsalis pedis artery, the posterior tibial artery was used.

Pulp skin blood flow was measured using laser Doppler fluxmetry (LDF; Periflux PF 4000, Perimed AB, Järfälla, Sweden), giving a semi-quantitative measurement of changes in cutaneous peripheral microcirculation expressed in arbitrary units (AU) [14]. After preparing the skin with an alcohol swab, we attached LDF probes (404–1; Perimed AB, Järfälla, Sweden) bilaterally to the pulps of the first toes.

We recorded arterial pressure continuously from the third finger of the right hand using a photoplethysmographic volume-clamp method (Finometer; FMS Finapres Medical Systems BV, Amsterdam, The Netherlands). Finometer pressures were calibrated with measurements from an automated sphygmomanometer (Solar 8000i, GE-Marquette Medical Systems, Inc., Milwaukee, USA) at the beginning of each experiment. Skin temperature was continuously measured within the vacuum chamber on the foot in close proximity to the dorsalis pedis artery using an Analog Devices AD590 temperature transducer (STR Teknikk, Aalesund, Norway).

Analog signals for all measurements were sampled at 300Hz and averaged for each heartbeat gated by the R-waves of the 3-lead ECG using custom-made software (REGIST 3). The patients were instructed to avoid moving and talking throughout the sampling period. All experiments were performed by the same researcher.

### Tolerability of negative pressure

At the end of each experiment, the patients rated their level of discomfort experienced during INP using a verbal numerical pain rating scale 0–10, ranging from zero (no pain or discomfort) to ten (worst imaginable pain).

### Statistical analysis

Patient characteristics are described by mean (standard deviation) or median (range). A mixed effects model was applied taking into account individual subject-to-subject variation. Mean differences and 95% confidence intervals (CI) were reported from the mixed effects model regression analysis. Statistical analyses were performed using R version 3.2.4 with the "nlme" [15] and "multcomp" [16] packages (R Foundation for Statistical Computing, Vienna, Austria). For all statistical tests, a 2-tailed probability level of *P*≤0.05 was considered statistically significant.

### Comparisons of mean values between sequences

For each subject, mean flow velocity (cm/s) and laser Doppler flux (AU) were calculated for each sequence (baseline, INP and post-INP). To remove disturbances in the transition zones, the first and last 10s of each sequence were removed before analysis. Effects of the INP and post-INP sequences compared to baseline were evaluated in a mixed effects regression model with subject as a random effect by assigning variables to each sequence.

### Changes over time within one negative pressure cycle

To examine the change in flow velocity over time within one negative pressure cycle, flow velocity was binned within each second after the onset of negative pressure (second 0 through 17), giving a total of 706 INP cycles for the 20 patients. The effect of each second after start of negative pressure compared to the mean flow velocity during the baseline sequence was evaluated in a mixed effects model with subject as a random effect by assigning variables to each second.

### Cumulative up-and-down fluctuations in arterial blood flow velocity

To evaluate the fluctuations in arterial blood flow velocity within the sequences, the differences between the flow velocity of each heartbeat and the previous were calculated. The sum of the absolute values of these differences were calculated within each sequence for each subject, and averaged per minute. This gave the average cumulative variation in blood flow velocity per minute. By assigning variables to the sequences, the effects of each sequence were calculated in a mixed effects model and estimates and confidence intervals calculated as above.

## Results

Twenty-one patients were included in the study. One patient was excluded to due inadequate, Doppler signals. As a result, 20 patients with PAD Fontaine stage grade II were included in the final analysis (Table 1). None of the patients used vasoactive drugs at the time of study. Blood flow velocity was measured in the dorsal pedis artery in all but two patients, where measurements were performed in the posterior tibial artery due to occluded dorsal pedis arteries.

**Table 1:** Patients’ characteristics, n = 20^†^.

Variables	
Age (yr)	75 (6.6)
Body mass (kg)	80 (12)
Height (cm)	174 (6.3)
BMI (kg/m^2^)	26 (3.4)
ABPI test leg	0.57 (0.18)
ABPI control leg	0.61 (0.21)
PVR test leg (mm)	5.2 (2.9)
PVR control leg (mm)	6.7 (3.8)
Systolic blood pressure (mmHg)	142 (23)
Diastolic blood pressure (mmHg)	72 (9)
Verbal Numerical Rating Pain Scale (0–10)	0 (0)
Maximal walking distance (m)	341 (107–1070)
Smoking (current / history / never), n (%)	6 (30%) / 13 (65%) / 1 (5%)
Diabetes mellitus (type 2), n (%)	4 (20%)
Statins, n (%)	18 (90%)
Platelet inhibitors, n (%)	19 (95%)
Prior revascularization in test leg, n (%)	12 (60%)

BMI, Body Mass Index; ABPI, Ankle-Brachial Pressure Index; PVR, Pulse Volume Recording

Values are mean (SD)

Values are median (min-max)

^†^18 males and 2 females

### Mean arterial blood flow velocity and mean laser Doppler flux

Compared to the baseline sequence, mean arterial blood flow velocity was higher during INP sequence and in the 5-min post-INP sequence (Table 2). There were no statistically significant effects of INP on mean laser Doppler flux in the control leg or test leg (Table 2).

**Table 2:** Physiological parameters for all the PAD patients (n = 20) during baseline, INP, and post-INP. Estimates are mean and 95% CI.

Parameters	Baseline		INP			Post-INP		
	Estimate	95% CI	Estimate	95% CI	*P*-value	Estimate	95% CI	*P*-value
Blood flow velocity, (cm/s)	6.7	5.2 to 8.3	7.5	5.9 to 9.1	0.03	7.5	5.9 to 9.0	0.035
LDF test leg (AU)	422	176 to 668	446	200 to 692	0.42	421	175 to 667	0.98
LDF control leg (AU)	646	388 to 903	659	402 to 918	0.66	662	404 to 920	0.62
Skin temperature (°C)	33.6	33.0 to 34.3	33.8	33.1 to 34.4	0.02	34.1	33.4 to 34.7	<0.001
MAP, (mmHg)	88	81 to 94	86	79 to 91	0.1	84	78 to 91	0.02
Heart rate (beats/min)	65	61 to 69	65	61 to 69	0.86	65	61 to 69	0.58

LDF, Laser Doppler flux; PAD, Peripheral Arterial Disease; INP, intermittent negative pressure; MAP, Mean arterial pressure; AU, Arbitrary Units

*P*-values are for comparisons to baseline

### Flow changes over time within one INP cycle

**Fig 3:**
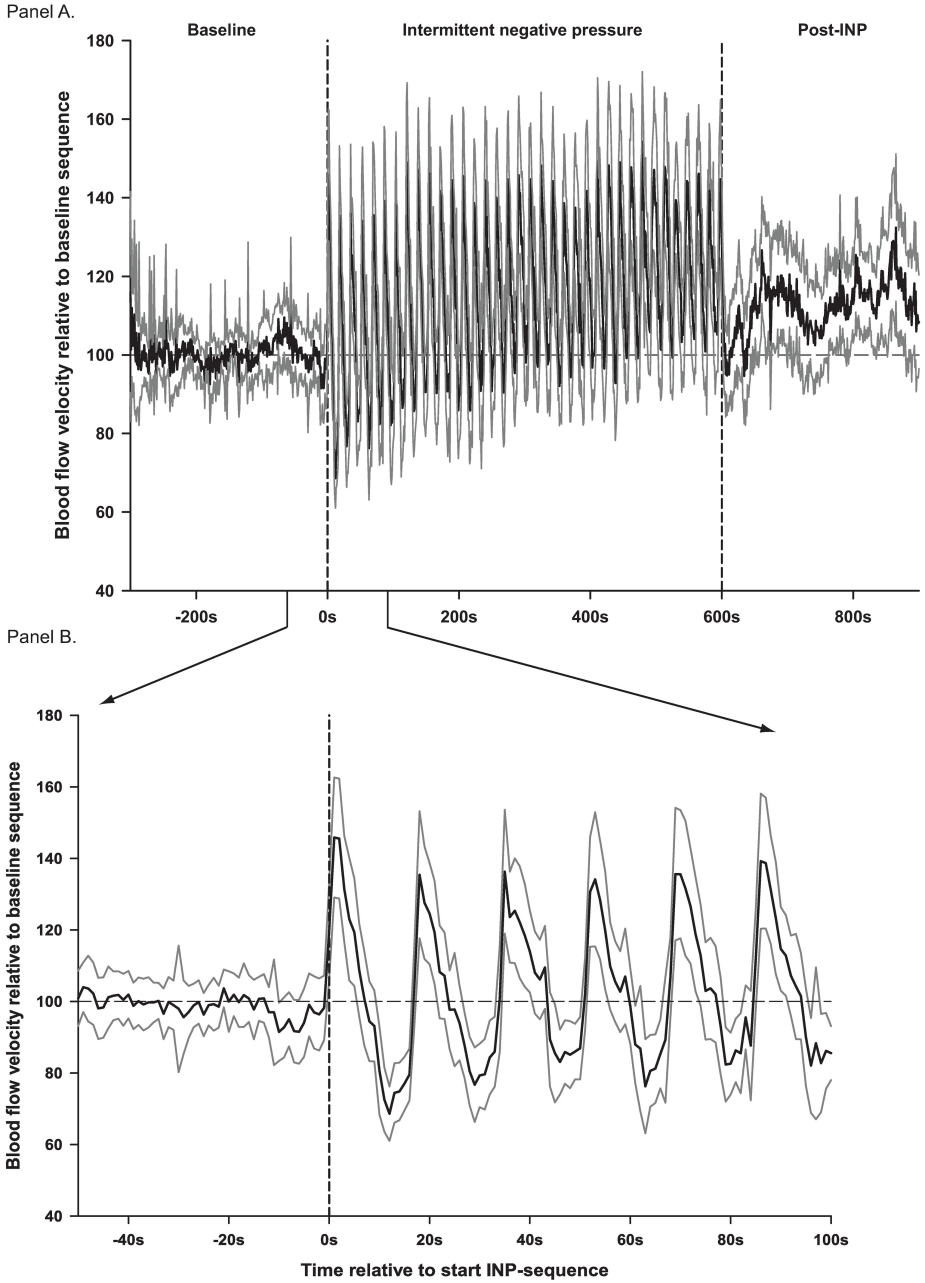
Arterial blood flow velocities (cm/s) for each second relative to each subject′s mean baseline value. Black lines are mean values with 95% confidence intervals for each second as grey lines. Panel A shows the whole 20-min experiment. Panel B shows a section at the end of baseline and beginning of INP.

**Fig 4:**
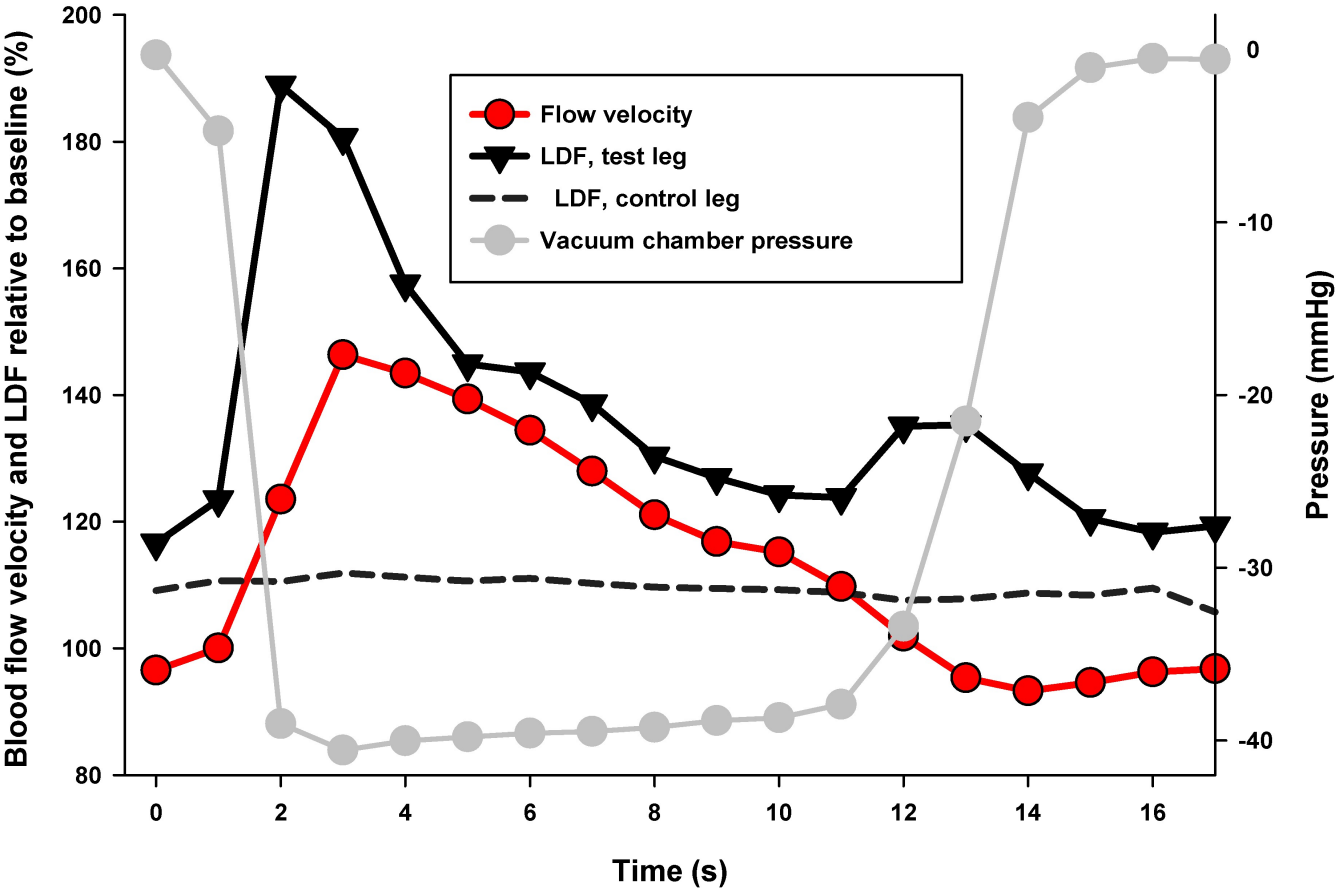
The effects of time for the first 17s (one pressure cycle) after onset of negative pressure. Effect estimates are for all the INP-cycles aggregated within and between subjects relative to each subject′s mean baseline value. Blood flow velocity (cm/s), Laser Doppler Flux, LDF (AU) measured in test leg and control leg (shown as dashed line); Vacuum chamber pressure (mmHg, right y-axis).

INP induced increase in blood flow velocity and flux pulsatility. The mean arterial blood flow velocity for each second during the INP sequence is presented in Fig 3. Estimates of changes in blood flow velocity (cm/s) and laser Doppler flux (AU) over time during one pressure cycle (17s) relative to the baseline sequence for all negative pressure cycles in all patients (706 pressure cycles analyzed in total) are presented in Fig 4. Peak arterial blood flow velocity was reached after 3s, with an increase of 46% (95% CI 36% to 57%, *P*<0.001) compared to baseline (Fig 4). Peak laser Doppler flux in the test leg was reached after 2s, with an increase of 89% [(95% CI 48% to 130%, *P*<0.001)] compared to baseline. Laser Doppler flux measured in the leg not exposed to INP (control leg) did not change during the pressure cycles [peak increase: 11% compared to baseline (95% CI -1% to 25%, *P*<0.08)] (Fig 4 and Table 2).

### Cumulative up-and-down changes in arterial blood flow velocity during INP

**Fig 5:**
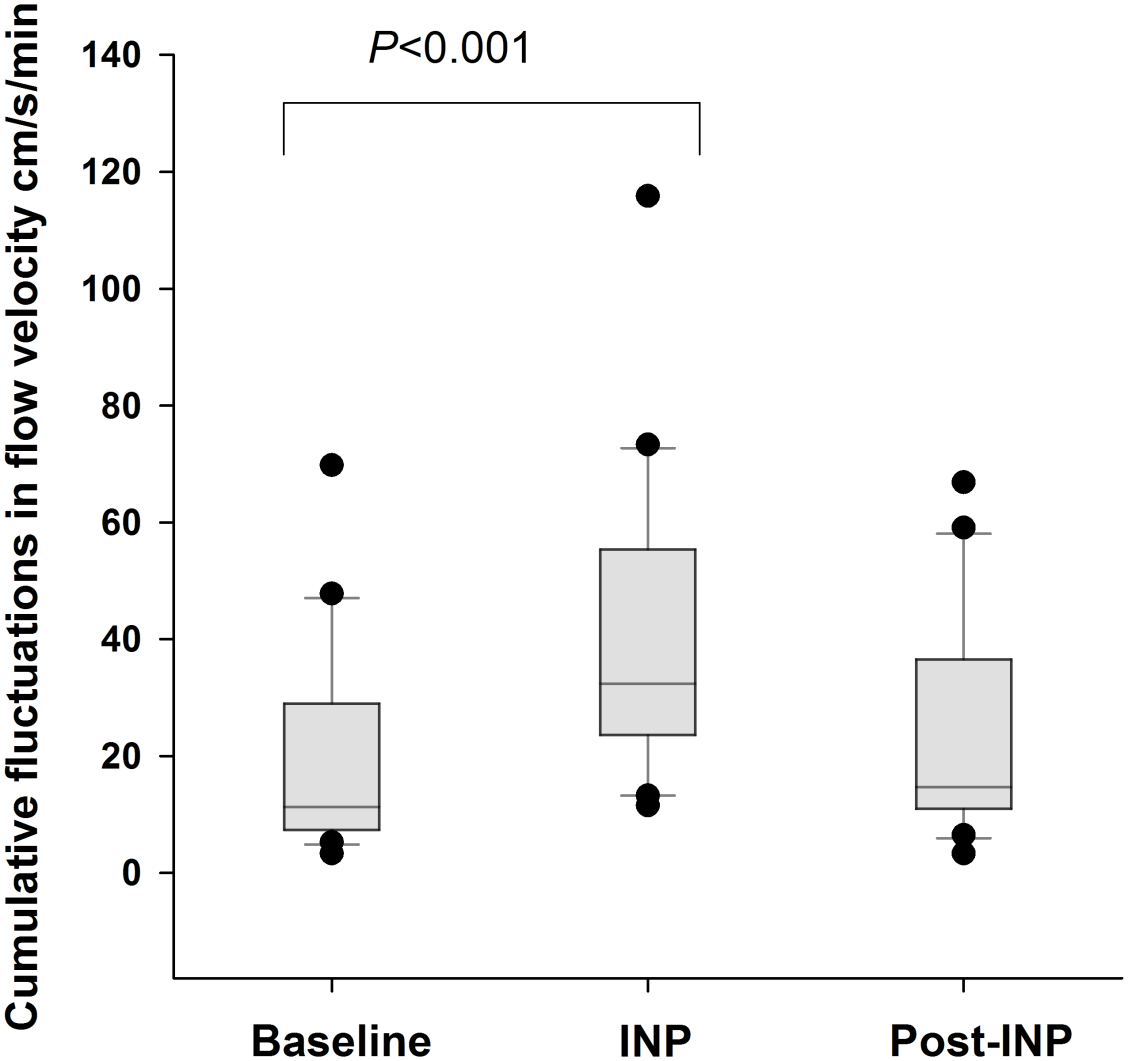
Fluctuations (cumulative up-and-down) in blood flow velocity during baseline (no pressure), INP, and post-INP (no pressure) sequences. The cumulative up-and-down are calculated per minute. Whiskers are 10^th^ and 90^th^ percentiles.

**Fig 6:**
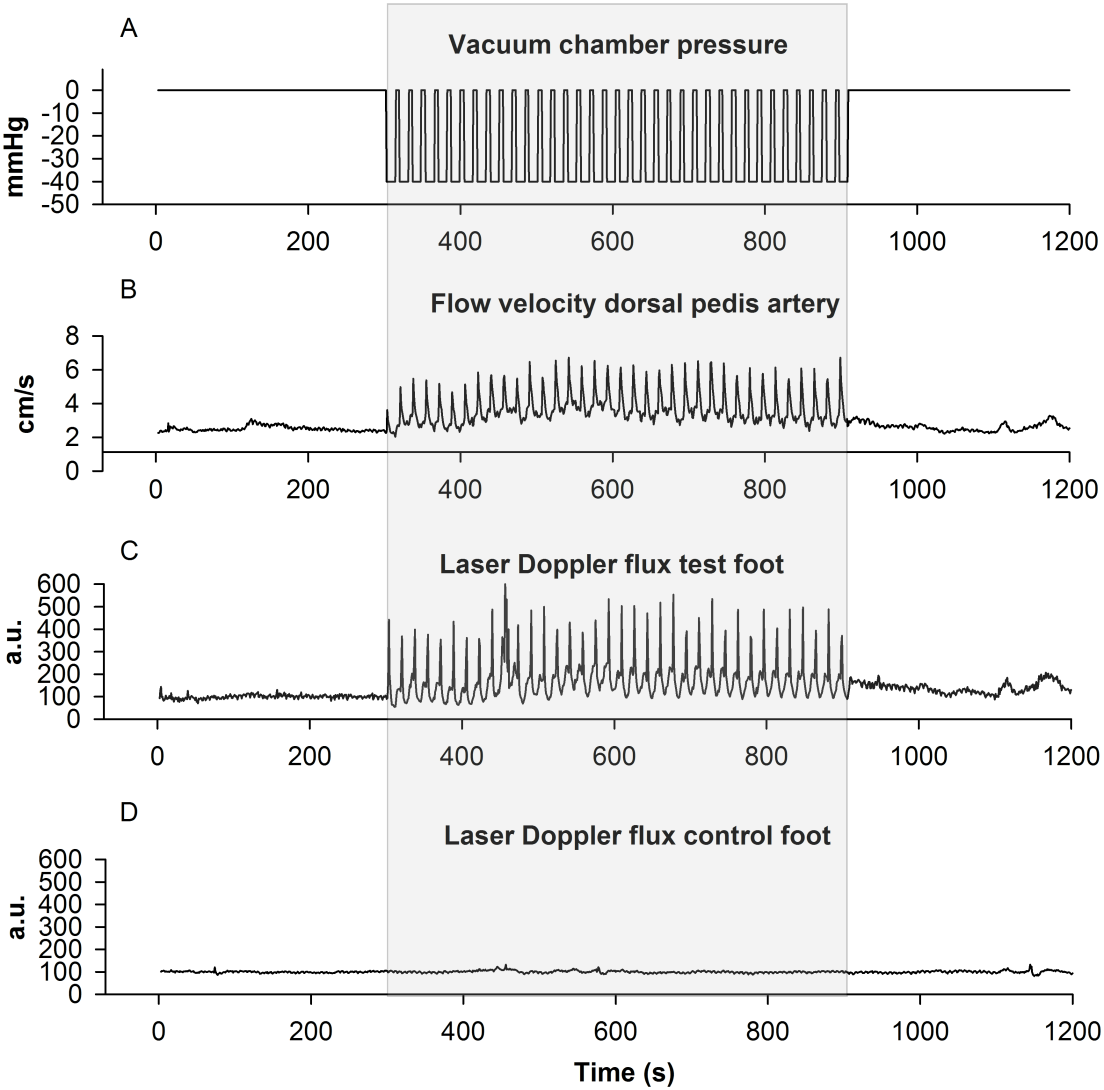
An example of blood flow velocity and laser Doppler flux responses in one of the patients (age: 64 years; Fontaine stage IIB; ABPI test leg: 0.21, ABPI control leg: 0.29, PVR test leg: 1mm, PVR control leg: 4mm) during the 20 minute experiment: 5 min baseline (atmospheric pressure), 10 min INP, and 5 min post-INP (atmospheric pressure) sequences. Panel A: Pressure within the vacuum chamber; Panel B: Blood flow velocity (ultrasound Doppler); Panel C: Laser Doppler flux in the test foot; Panel D: Laser Doppler flux in the control foot not exposed to INP. All panels’ timelines are from 0 to 1200s (x-axis).

Mean cumulative fluctuations were 21 (95% CI 12 to 30)cm/s/min in the baseline sequence. This increased to 41 (95% CI 32 to 51, *P*<0.001 compared to baseline)cm/s/min during the INP sequence. The post-INP sequence was not significantly different from baseline, 24 (95% CI 15 to 33, *P* = 0.38 compared to baseline)cm/s/min. Cumulative fluctuations during the sequences are presented in Fig 5, and blood flow velocity and flux responses in one patient during the whole 20 minute experiment are presented in Fig 6.

### Central hemodynamics—Heart rate and blood pressure

Heart rate and blood pressure are presented in Table 2. There were no clinically significant differences in mean arterial pressure (MAP) or heart rate (HR) during between the INP or post-INP sequences compared to baseline.

## Discussion

The main finding in the present study was that applying INP (-40mmHg) to the lower leg and foot in PAD patients increased fluctuations in arterial blood flow velocity compared to baseline (Figs 3 and 4). INP induced an increase in peak arterial blood flow velocity of 46% at the onset of negative pressure compared to average baseline blood flow velocity (S1 Video). A corresponding increase in pulp skin blood flow fluctuations in the test foot was observed during INP (Fig 4), while skin blood flow on the foot outside the vacuum chamber did not change (Fig 4). The cumulative fluctuations in arterial blood flow velocity during the INP sequences were significantly higher compared to the baseline sequences (Fig 5). This fluctuation response reflects increased blood flow pulsatility during INP compared to baseline (Figs 3 and 4).

The proposed theoretical foundation for applying negative pressure intermittently instead of constantly is that constant negative pressure applied to a body part causes venous distension and reduces blood flow locally via the venoarteriolar reflex [17, 18]. Accordingly, we recently demonstrated that constant negative pressure of -40mmHg applied to the lower leg and foot resulted in a decreased arterial blood flow velocity, skin blood flow, and skin temperature in the foot of healthy volunteers [8]. The same negative pressure applied intermittently resulted in increased blood flow pulsatility and an increase in mean blood flow velocity [8]. In these healthy volunteers, we found that INP increased peak blood flow velocity in foot arteries by 44% during the first few seconds after onset of negative pressure [8]. The present study on PAD patients observed that blood flow pulsatility increased as seen in the study on healthy volunteers [8]. This shows that the peripheral vasculature in the PAD patients may have a comparable ability to increase macro- and microcirculatory foot circulation during INP as healthy subjects.

In order to regulate vascular tone and tissue perfusion, endothelial cells sense blood flow and react to this mechanical stimulus, releasing vasoconstriction and vasodilation substances [19]. Considering the effects of flow shear stress on the release of biochemical mediators such as prostacyclin [20] and endothelial-derived substances [21–23], INP-induced flow pulsatility may cause beneficial vascular adaptions after repetitive use. Compared to nonpulsatile flow conditions, endothelium-derived nitric oxide release is significantly enhanced during conditions of flow pulsatility in the peripheral vasculature in vivo [24], and in vitro using cell cultured endothelial cells [25]. The relatively modest (~12%) increase in mean arterial blood flow velocity during INP sequences in the PAD patients (Table 2) is similar to that observed in healthy volunteers (~8%) [8]. This finding is somewhat surprising given the marked increase in blood flow pulsatility (up-and-down fluctuations) during INP, as shown in Figs 3 and 4. These blood flow velocity fluctuations caused the blood flow velocity, after reaching a maximum at about 3-4s after onset of negative pressure, to gradually decline below baseline values after about 12s (Fig 4). This decline in blood flow velocity below baseline values will reduce the mean blood flow velocity during the INP sequence. This gradual reduction in blood flow velocity relative to baseline described in Figs 3 and 4 may be due to venous distension, which elicits the venoarteriolar reflex [17, 18]. Alternatively, the decline in blood flow velocity may be due to a decreased pressure gradient, which is in turn caused by continued arterial inflow during the negative pressure cycles [9].

In the present study, there was also a significant (~12%) increase in mean blood flow velocity during the 5-min post-INP sequence compared to baseline (Table 2 and Fig 3, panel A), suggesting that INP had a prolonged effect on blood flow. This increase in post-INP blood flow velocity was observed despite no significant increase in cumulative up-and-down fluctuations in blood flow velocities compared to baseline (Fig 5). This increase in mean arterial blood flow velocity during the post-INP sequence may be due to INP-induced shear stress. Flow-mediated vasodilation may therefore explain some of the positive clinical outcomes after INP treatment reported in earlier studies [26–29], and our own observations [9]. The potential impact of long-term INP use on flow-mediated vasodilation should be the subject of further studies.

Reports on "suction devices" to the lower limb for the treatment of tissue ischemia date back to the early 19^th^ century [26, 30–35]. These initial studies were limited by the lack of objective outcome variables (i.e. use of self-reported walking distance), small sample sizes, and weak study designs [36, 37]. In the late 1960s, Smyth [27] performed the first analyses of the isolated effects of INP (-150mmHg) on femoral artery flow. This study reported data on acute blood flow measurements in femoral arteries and veins, as well as calf inflow in patients with peripheral vascular disease and intermittent claudication. Smyth [27] used plethysmography to measure calf inflow in 11 patients following 6 weeks of INP treatment two times per week, reporting a 282% increase in mean resting blood flow in the calves, and a 300% increase in peak reactive hyperemia after 2 minutes of occlusion. This study measured arterial blood flow velocity changes during INP, but did not provide any data on INP’s effect on foot circulation, skin blood flow or central hemodynamics [27]. The protocol Smyth [27] described was later replicated by Gill et al. [38] in a four week intervention study on three patients with Raynaud’s disease of the hands and eight patients with intermittent claudication. Gill et al. [38] reported increased systolic ankle pressure and calf muscle blood flow (133^XE^ clearance technique) in claudicants, as well as increased hand skin blood flow in the patients with Raynaud’s disease.

In a randomized crossover trial, Himmelstrup et al. [28] reported beneficial effects of intermittent negative and positive pressure applied to the lower body, with pressure oscillations lasting 5-10s (Vacusac, Intl. aps, Denmark). In this study, 22 patients with intermittent claudication were randomized to either 2 months Vacusac (active treatment) or placebo. The main findings were that patients receiving active treatment significantly increased pain-free and maximal walking distance on the treadmill and increased their ABPIs [28]. Importantly, the increased ABPIs were followed by a decrease in systemic blood pressure [28]. When patients were crossed over, those receiving active treatment again improved, while the placebo group did not. Similar improvements in ABPIs, pain-free and maximal walking distance on 34 patients with intermittent claudication have also been described by Mehlsen et al. [29] in a double-blinded randomized trial comparing 25 Vacusac treatments to 25 placebo treatments over two months. Mehlsen et al. [29] also reported significant increases in the ADP threshold for platelet aggression after treatment.

In a recent case study on four PAD patients (ABPI ≤0.60) with long-lasting (6–24 months) hard-to-heal leg ulcers, we observed—in addition to improved wound healing—similar results to those of the aforementioned Himmelstrup and Mehlsen studies [28, 29] on foot perfusion. We observed increases in ABPIs after eight weeks of INP therapy two hours per day [9]. Our findings on the acute effect of INP on increased macro- and microcirculatory circulation in the present study on PAD patients may explain the clinically beneficial effect of ambient pressure therapy applied to the lower limb on wound healing and ischemic limbs, consistent with the aforementioned studies.

In contrast to the commonly used negative pressure wound therapy (NPWT), which applies high negative pressure (-50 to -125mmHg) to the local wound environment to remove excessive fluid [39], the INP method used in the present study applies mild negative pressure to a large area of the lower limb (Fig 2). This could theoretically facilitate increased arterial inflow in all six angiosomes of the lower leg and foot due to mechanical dilation of the tissue and vessels exposed to INP. In contrast to the findings that INP resulted in increased flow pulsatility in arteries and the small vessels distally in the foot in patients with PAD, NPWT has shown to decrease blood flow in close proximity to the negative pressure area [40]. Additionally, whether NPWT increases tissue perfusion remains controversial [41, 42].

### Limitations of the study

There are some limitations to the present study. Firstly, blood flow velocities were used to describe changes in arterial flow in the foot in PAD patients. We were not able to measure the diameter of the dorsalis pedis and tibial posterior arteries within the vacuum chamber during INP. However, pulsatile diameter in small arteries has been found to be very stable [43]. We therefore believe it is reasonable to assume that the changes observed in blood flow velocity during INP reflect changes in blood flow in the foot artery. If the vessel diameter increased during negative pressure, then our measurements would underestimate arterial blood flow velocity during INP.

Secondly, skin temperature was measured on the dorsum of the foot, and the temperature sensor was isolated from the surrounding air with several layers of adhesive tape (Micropore Surgical Tape, 3M, MN, US). It is possible that the tape did not adequately isolate the probe and that an increase in skin temperature could be due to increased temperature inside the "air pocket" within the vacuum chamber. Leakage results in different pressure curves, which could give a different physiological response. Nevertheless, we observed increased skin temperature together with increased peak blood flow velocity and laser Doppler flux during the pressure cycles. Skin blood flow measured with laser Doppler flux has been found to be highly correlated with local skin temperature in subjects exposed to temperatures between 23 to 36°C (r = 0.87) [44]. Together, these findings indicate increased tissue perfusion of the foot during INP.

Thirdly, our study had an unequal number of males and females. Differences between sexes in the INP-induced flow responses may be a subject for further studies.

## Conclusions

In the present study on patients with PAD, we observed increased peripheral blood flow pulsatility in the foot during application of INP to the lower limb. The mean arterial blood flow velocity increased slightly during INP and the increase sustained for at least a 5-min period after termination of INP. The increased laser Doppler flux and skin temperature observed during the negative pressure cycles reflect increased blood flow to the small vessels of the skin. The observed increase in macro- and microvascular flow during INP in patients with ischemic limbs may improve tissue perfusion, wound healing and patency after revascularization. Future studies are warranted to explore the working mechanisms of INP and possible clinically relevant effects after repetitive exposure to INP.

## Supporting information

S1 Video A live Doppler signal response during intermittent negative pressure applied to the lower leg and foot in a patient with peripheral arterial disease (Fontaine stage II). The video shows a typical live Doppler signal response when measuring blood flow velocity in the most distal foot artery (*arteria dorsalis pedis*). Twenty seconds into the recording, the negative pressure is applied for 10 second followed by a 7 second period of atmospheric pressure. The sequence is repeated with the negative pressure (-40 mmHg) starting again after 37 seconds and 54 seconds.
(MP4)

S1 Data The dataset includes all of the 20 PAD patients. (TXT)
